# Delivery of Iron-Sulfur Clusters to the Hydrogen-Oxidizing [NiFe]-Hydrogenases in *Escherichia coli* Requires the A-Type Carrier Proteins ErpA and IscA

**DOI:** 10.1371/journal.pone.0031755

**Published:** 2012-02-21

**Authors:** Constanze Pinske, R. Gary Sawers

**Affiliations:** Institute for Biology/Microbiology, Martin-Luther University Halle-Wittenberg, Halle (Saale) Germany; Auburn University, United States of America

## Abstract

During anaerobic growth *Escherichia coli* synthesizes two membrane-associated hydrogen-oxidizing [NiFe]-hydrogenases, termed hydrogenase 1 and hydrogenase 2. Each enzyme comprises a catalytic subunit containing the [NiFe] cofactor, an electron-transferring small subunit with a particular complement of [Fe-S] (iron-sulfur) clusters and a membrane-anchor subunit. How the [Fe-S] clusters are delivered to the small subunit of these enzymes is unclear. A-type carrier (ATC) proteins of the Isc (iron-sulfur-cluster) and Suf (sulfur mobilization) [Fe-S] cluster biogenesis pathways are proposed to traffic pre-formed [Fe-S] clusters to apoprotein targets. Mutants that could not synthesize SufA had active hydrogenase 1 and hydrogenase 2 enzymes, thus demonstrating that the Suf machinery is not required for hydrogenase maturation. In contrast, mutants devoid of the IscA, ErpA or IscU proteins of the Isc machinery had no detectable hydrogenase 1 or 2 activities. Lack of activity of both enzymes correlated with the absence of the respective [Fe-S]-cluster-containing small subunit, which was apparently rapidly degraded. During biosynthesis the hydrogenase large subunits receive their [NiFe] cofactor from the Hyp maturation machinery. Subsequent to cofactor insertion a specific C-terminal processing step occurs before association of the large subunit with the small subunit. This processing step is independent of small subunit maturation. Using western blotting experiments it could be shown that although the amount of each hydrogenase large subunit was strongly reduced in the *iscA* and *erpA* mutants, some maturation of the large subunit still occurred. Moreover, in contrast to the situation in Isc-proficient strains, these processed large subunits were not membrane-associated. Taken together, our findings demonstrate that both IscA and ErpA are required for [Fe-S] cluster delivery to the small subunits of the hydrogen-oxidizing hydrogenases; however, delivery of the Fe atom to the active site might have different requirements.

## Introduction

Iron-sulfur ([Fe-S]) clusters are ubiquitous prosthetic groups of many metalloenzymes in almost all life-forms and they have a variety of functions in diverse cellular processes. Generation of [Fe-S] clusters does not occur spontaneously but requires dedicated machineries that orchestrate their assembly and subsequent transfer to apoprotein substrates (for reviews see [1]–[3]. There are at least three different [Fe-S] biosynthetic systems known and they are referred to as Nif (nitrogen fixation-associated), Isc (iron sulfur cluster) and Suf (sulfur mobilization). The initial discovery of the specialized NifUS proteins for the generation of [Fe-S] clusters in the nitrogenase enzyme of the nitrogen-fixing bacterium *Azotobacter vinelandii* [4] made it immediately clear that further generalized [Fe-S] machineries in bacteria must exist and these are represented by the Isc and Suf systems in many microbes [5], [6].

The protein components of the Isc and Suf biogenesis systems can be roughly divided into those proteins dedicated to [Fe-S] assembly and those proposed to be involved in the subsequent trafficking of the pre-formed cluster to the ultimate apoprotein acceptor [3]. The proteins involved in transfer or trafficking of [Fe-S] are referred to as A-type carrier (ATC) proteins and the bacterium *Escherichia coli* has three of these, which are phylogenetically related [7], and are termed IscA, SufA and ErpA [8]. Current evidence is consistent with a role in cluster transfer between the Isc or Suf scaffold machinery and apoprotein substrates [3], [9], [10]; however, it has also been proposed that the ATC proteins deliver iron to the scaffold proteins [11].

An *erpA* mutation severely impairs aerobic growth of *E. coli* while single mutations in the *sufA* and *iscA* genes are viable [8]. Individual knock-out mutations in the *iscA, erpA* or *sufA* genes have a limited effect on anaerobic growth [8], suggesting redundancy of function for [Fe-S] cluster insertion into key iron-sulfur proteins. This contrasts with *iscA sufA* double null mutants, which generally are non-viable aerobically unless another means of [Fe-S] cluster assembly is present, such as heterologously expressed *nifUS*, or introduction of the eukaryal isoprenoid biosynthetic pathway [3], [7], [12]. Similarly, *iscA erpA* double mutants also require introduction of the eukaryal isoprenoid biosynthetic pathway together with supplementation of mevalonate to restore growth [7], [8]. Taken together these genetic studies suggest that IscA and SufA are functionally redundant during aerobic growth of *E. coli*, while IscA and ErpA show redundancy even anaerobically. Little is known, however, about which apoproteins these putative [Fe-S] cluster-delivery proteins have as substrates.

**Figure 1:**
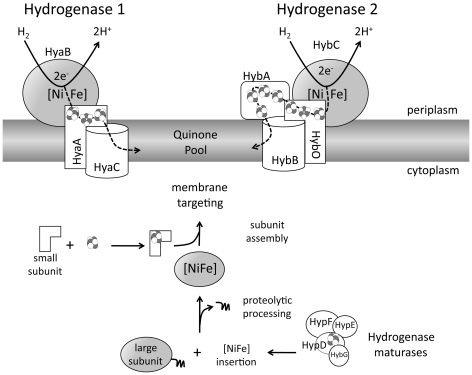
Schematic representation of the iron-sulphur cluster-containing proteins involved in the hydrogen oxidation of *E. coli*. The two anaerobic, membrane-associated [NiFe]-hydrogenases 1 and 2 are schematically represented with their associated subunits. The iron-sulphur clusters are shown as groups of spheres. No distinction is made between [3Fe-4S], [4Fe-4S] or [4Fe-3S] clusters. The ‚squiggle' attached to the apoprotein form of the hydrogenase catalytic subunit (bottom of the Figure) represents the C-terminal peptide that is removed subsequent to insertion of the [NiFe] cofactor. The dotted arrows indicate electron flow within the modular enzymes or to the quinone pool.

[NiFe]-hydrogenases are evolutionarily ancient [Fe-S] cluster-containing proteins that catalyze the reduction of protons to molecular hydrogen or the oxidation of hydrogen to protons and electrons [13], [14]. The genome of *E. coli* encodes four membrane-associated [NiFe]-hydrogenases, only three of which are synthesized under anaerobic growth conditions. Hydrogenase 1 and hydrogenase 2 have their respective active site located in the periplasm and they both catalyze hydrogen oxidation (Fig. 1). Hydrogenase 3 forms part of the multi-subunit, hydrogen-evolving formate hydrogenlyase (FHL) complex [15], which disproportionates formic acid into CO_2_ and H_2_ during fermentation.

The two hydrogen-oxidizing hydrogenases comprise a large catalytic subunit (HyaB and HybC for hydrogenase 1 and 2, respectively; see Fig. 1), which lacks a [Fe-S] cluster but has a [NiFe] cofactor that catalyzes hydrogen activation, a small, electron-transfer subunit with three [Fe-S] clusters (HyaA and HybO for hydrogenase 1 and 2, respectively), as well as a membrane-anchor subunit that carries menaquinone-binding sites (HyaC and HybB, for hydrogenase 1 and 2, respectively) [15]–[18]. Hydrogenase 2 has an additional [Fe-S] cluster subunit, HybA, which is not a typical small subunit, but has been shown to be required for growth on hydrogen and fumarate [19] (Fig. 1).

Synthesis and assembly of these modular, cofactor-containing hydrogen-oxidizing hydrogenases require the coordinated involvement of a number of ancilliary proteins. For example, specific hydrogenase maturases, termed Hyp proteins, are required to synthesize and insert the [NiFe] cofactor into the apo-form of the large subunit [13], [15]. Once synthesis and insertion of the [NiFe] cofactor has been completed, the large subunit is processed at its C-terminus by a hydrogenase-specific endopeptidase. Only after this step can the mature large subunit associate with the mature, [Fe-S] cluster-containing small subunit. The respective small subunits of hydrogenases 1 and 2 have an N-terminal Tat (twin-arginine transport) signal sequence, which directs the dimeric protein to the membrane where it is transported across the cytoplasmic membrane by the Tat-translocon [20]. After transport the dimer of the large and small subunits associates with the respective membrane-integral anchor subunit to generate an active hydrogen-oxidizing hydrogenase.

The involvement of [Fe-S] clusters in the biosynthesis and activity of the hydrogen-oxidizing [NiFe]-hydrogenases is extensive and is summarized in Fig. 1. While a considerable amount of information is available concerning the biosynthesis of the [NiFe]-center and the roles of the Hyp maturases (for reviews see [13], [15]), as well as the route of nickel incorporation [21], virtually nothing is known about either the routes of incorporation of the [Fe-S] clusters into the small subunits or where the iron atom in the active site originates. In this study, we have examined the biosynthesis of the hydrogen-oxidizing [NiFe]-hydrogenases of *E. coli* with respect to the potential involvement of the three ATC paralogues IscA, SufA and ErpA. Our results reveal that IscA and ErpA are both essential for the assembly of active hydrogen-oxidizing hydrogenases.

## Results

### The iron-sulphur cluster trafficking proteins IscA and ErpA are both required for hydrogen-oxidizing enzyme function

**Figure 2:**
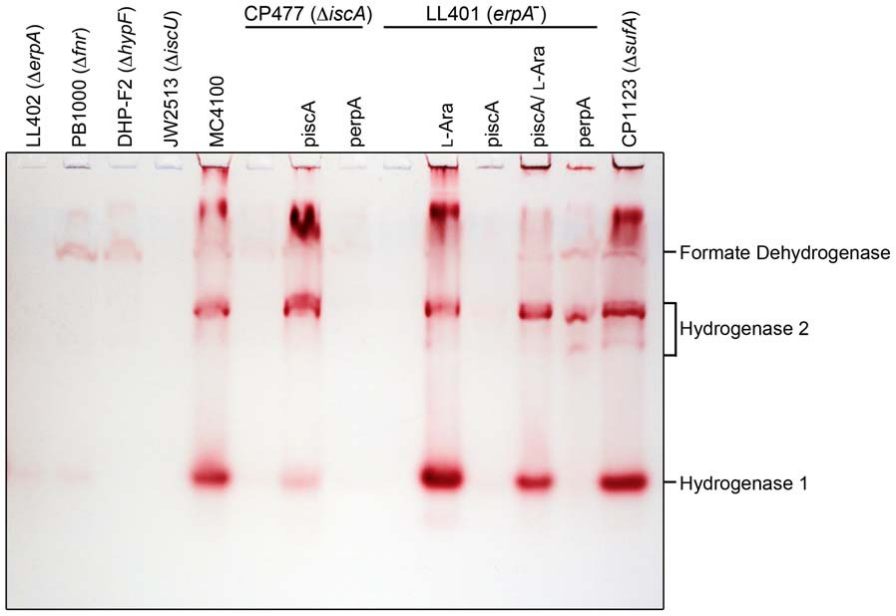
Hydrogenases 1 and 2 are inactive in *iscA* and *erpA* mutants. Aliquots of crude extracts (25 µg protein) derived from the bacterial stains shown were separated by non-denaturing PAGE (7.5% w/v polacrylamide) and subsequently stained for hydrogenase enzyme activity. Hydrogenase 1 migrates as a single active enzyme species while hydrogenase 2 shows multiple active forms, which are designated on the right of the panel. The weak hydrogen: benzyl viologen oxidoreductase activity that is independent of the [NiFe]-hydrogenases, and which is associated with formate dehydrogenase [25], is also designated.

Initially we wanted to examine the effects of deleting the genes encoding the ATC proteins IscA, SufA and ErpA on hydrogen-oxidizing enzyme function in anaerobic *E. coli* cells. The activities of hydrogenases 1 and 2 can be readily identified after separation in native PAGE followed by specific hydrogenase activity-staining, thus providing a facile method of exclusively examining the consequences of mutations on hydrogen-oxidizing enzyme activity [22], [23]; hydrogenase 3 enzyme activity is labile under these conditions and therefore does not interfere with this analysis. Extracts derived from a *sufA* mutant revealed hydrogenase 1 and hydrogenase 2 activity profiles similar to the hydrogenase wild type strain MC4100 (Fig. 2). In contrast, extracts derived from the *iscA* and *erpA* mutants were devoid of both activities. Both strains retained a slowly migrating hydrogen: benzyl viologen oxidoreductase activity that has been shown to be due to a side reaction of the respiratory formate dehydrogenases [24], [25]. As a further control, we analyzed the hydrogenase 1 and hydrogenase 2 activity profiles in the *iscU* mutant JW2513 and observed no activity of either enzyme (Fig. 2). In general, this is a similar phenotype to that of the Δ*hypF* mutant DHP-F2, which cannot synthesize the [NiFe] cofactor and therefore lacks all hydrogenase enzyme activities [26]. This phenotype is also similar to that observed in a mutant lacking the transcriptional regulator Fnr (Fig. 2), which lacks hydrogenase 1 and 2 activities [23], [27].

Quantitative determination of total hydrogenase enzyme activity in the crude extracts from these mutants revealed that when compared with extracts derived from the hydrogenase strain MC4100, the activity was reduced by more than 95% in the *iscA* and *erpA* mutants and was abolished in the *iscU* mutant (MC4100, 3.00±0.59 U mg protein^−1^; CP477 (*iscA*), 0.12±0.07 U mg protein^−1^; LL401 (*erpA*), 0.13±0.07 U mg protein^−1^; JW2513 (*iscU*), <0.01 U mg protein^−1^). The residual hydrogenase enzyme activity measured in the *iscA* and *erpA* mutants was due to hydrogenase 3 (data not shown). Taken together, these data demonstrate that the hydrogen-oxidizing hydrogenases 1 and 2 depend on both ErpA and IscA for enzyme activity. Furthermore, the Isc machinery, but not the Suf machinery, is required for hydrogen oxidation in *E. coli*.

**Figure 3:**
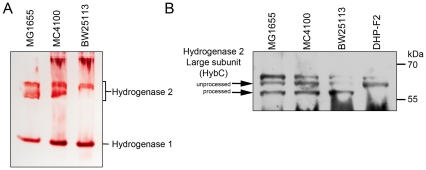
Comparative analysis of hydrogen-uptake hydrogenases in different *E. coli* K-12 derivatives. A. Aliquots of crude extracts (25 µg protein) derived from the bacterial strains shown were separated by non-denaturing PAGE (7.5% w/v polyacrylamide) and subsequently stained for hydrogenase enzyme activity. The locations of hydrogenase 1 and hydrogenase 2 activity bands are shown. B. Western blot analysis of the unprocessed and processed forms of the HybC large subunit of hydrogenase 2 was performed using crude extracts (25 µg protein) derived from the bacterial strains indicated. Strain DHP-F2 is a derivative of MC4100 carrying a deletion in the *hypF* gene.

It should be noted that various *E. coli* K-12 wild type strains, including MC4100, MG1655 and BW25113 and mutants thereof, shared the same hydrogen-oxidizing phenotype and are therefore directly comparable (Fig. 3). The total hydrogenase activities were 2.80±0.48 U mg protein^−1^ for MG1655 and 2.74±0.43 U mg protein^−1^ for BW25113.

### Complementation analysis

Introduction of the *iscA* gene on a multicopy plasmid (piscA) into the *iscA* mutant CP477 restored total hydrogenase enzyme activity (3.59±0.19 U mg protein^−1^) to levels similar to those in MC4100. Similarly, introduction of the *erpA* gene on plasmid perpA restored total hydrogenase activity to the conditional *erpA* mutant LL401 (3.37±1.45 U mg protein^−1^). Because the *erpA* gene in LL401 is under the control of the *ara_P_* promoter [8], growth of the strain in the presence of arabinose induces *erpA* expression and restored hydrogenase activity (5.34±1.02 U mg protein^−1^). The reason why the activity in the presence of arabinose was significantly higher than in the wild type is currently unclear.

Analysis of extracts derived from CP477 and LL401 transformed with either piscA or perpA revealed that only multicopy *iscA* restored hydrogenase 1 and hydrogenase 2 activities to CP477, while only growth in the presence of arabinose (or less effectively perpA) restored hydrogenase 1 and hydrogenase 2 activities to LL401 (Fig. 2); addition of L-arabinose to strain LL401 carrying plasmid piscA was performed as a control to demonstrate that multicopy *iscA* did not interfere with complementation by induced *erpA* expression. The *erpA* gene when supplied in *trans* on a plasmid was less effective at restoring hydrogenase 1 activity to strain LL401 and the reason for this is unclear; however, it suggests that hydrogenase 1 and hydrogenase 2 might be differentially responsive to the cellular levels of ErpA. The recovery of total hydrogenase by plasmid perpA in LL401 can be explained by the fact that under the growth conditions analyzed here hydrogenase 3 comprises the bulk of the measureable activity with a limited overall contribution by hydrogenase 1 [23], [24].

### Low levels of the processed hydrogenase catalytic subunit in *iscA* and *erpA* mutants

**Figure 4:**
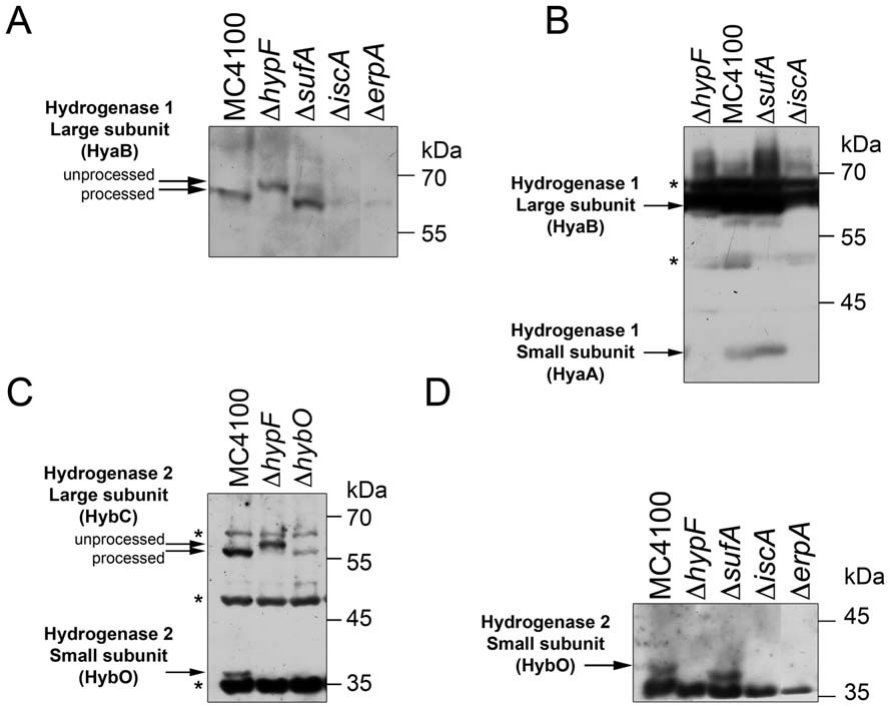
Immunological analysis reveals maturation of the catalytic subunit and absence of the small subunit of the hydrogen-uptake enzymes in *erpA* and *iscA* mutant. Samples of crude extracts (25–50 µg protein) derived from the strains (genotypes are shown above each lane) indicated were separated in 10% (w/v) SDS-PAGE, transferred to nitrocellulose membranes and probed with antibodies against the hydrogenase 1 large subunit (HyaB) (A) or the small subunit (B). The locations of the processed and unprocessed forms of the large subunit polypeptide are shown on the left of the panel and the migration position of molecular mass size markers are shown on the right. Results of a similar experiment showing western blots of the large (HybC) and small (HybO) subunits of hydrogenase 2 are shown in C and D. Unspecific cross-reacting polypeptides that were used as internal loading controls are marked with an asterisk. MC4100, wild type; DHP-F2 (Δ*hypF*); CP1223 (Δ*sufA*); CP477 (Δ*iscA*); LL402 (Δ*erpA*); CP795 (Δ*hyaB* Δ*hybO* Δ*hycE*) was used in the lane labelled Δ*hybO*.

It is possible to distinguish two forms of the hydrogenase catalytic subunits using western blot analysis after SDS-PAGE [13]. The two forms of the approximately 65 kDa large subunit represent an unprocessed polypeptide and a processed form of the polypeptide. The processed species lacks a short C-terminal peptide that results from a specific endoproteolytic cleavage event [13], [28] and which only occurs after insertion of the [NiFe] cofactor has been completed [29]. Western blot analysis of the catalytic subunit (HyaB) of hydrogenase 1 in crude extracts derived from the strain MC4100 revealed mainly the faster-migrating, processed polypeptide (Fig. 4A). In contrast, a mutant unable to make the HypF maturase, which provides the cyanide ligands to the iron atom of the [NiFe] cofactor [26], showed only the unprocessed form of the polypeptide (Fig. 4A) because the [NiFe] cofactor cannot be synthesized. Analysis of the hydrogenase 1 large subunit in a crude extract derived from the *sufA* mutant CP1223 revealed mainly the processed form at a level similar to that observed for MC4100 (Fig. 4A). This is consistent with the wild type level of hydrogenase 1 activity seen in the *sufA* mutant (see Fig. 2). Analysis of extracts derived from the *iscA* and *erpA* mutants revealed that there were very low amounts of the processed form of the polypeptide in the mutants (Fig. 4A). This finding shows that, despite the reduced level of the protein, possibly due to enhanced degradation, the reason for the lack of detectable hydrogenase 1 enzyme activity in Fig. 2 was not because the catalytic subunit could not receive the completed [NiFe] cofactor. Similar observations were made for the catalytic subunit (HybC) of hydrogenase 2 (see below and Fig. 4).

### The respective [Fe-S] cluster-containing small subunit of both hydrogen-uptake hydrogenases is absent in a *hypF* mutant

The antibodies raised against hydrogenase 1 and hydrogenase 2 also recognize the respective [Fe-S] cluster-containing small subunit. Western blot analysis of crude extracts derived from MC4100 grown under fermentative conditions using antiserum raised against hydrogenase 1 (Fig. 4B) or hydrogenase 2 (Fig. 4C) identified the small subunits HyaA and HybO, respectively, each with a molecular mass of approximately 35 kDa [30], [31]. As a control, no hydrogenase 2 small subunit (HybO) could be detected in extracts of the *hybO* mutant CP795 (Fig. 4C). Notably, the level of the large subunit (HybC) was significantly reduced in CP795 compared with extracts of MC4100, which can synthesize an active hydrogenase 2. This suggests that in the absence of the small subunit, the processed large subunit is more rapidly degraded.

In extracts derived from the Δ*hypF* strain DHP-F2 only the unprocessed form of the large subunits of hydrogenases 1 and 2 were detected (Fig. 4A and C). Surprisingly, however, the respective HyaA and HybO small subunits could not be detected (Fig. 4B, C). This suggests that in the absence of the processed form of the catalytic subunit, the small subunit is subject to rapid degradation. This can be concluded because the genes encoding the respective small subunits are the first genes in the multicistronic *hya* and *hyb* operons [17], [18], thus ruling out a transcriptional effect.

### The respective [Fe-S] cluster-containing small subunit of both hydrogen-uptake hydrogenases is absent in *iscA* and *erpA* mutants

Recent studies have demonstrated that the [Fe-S] cluster-containing small subunit is essential to allow measurement of hydrogen-dependent benzyl viologen (BV) reduction of the hydrogenase 1, hydrogenase 2 and hydrogenase 3 enzymes [24]. Western blot analysis of extracts derived from the *iscA* mutant CP477 and the *erpA* mutant LL402 [8] also lacked the small subunits of hydrogenase 2 (Fig. 4D) and hydrogenase 1 (Fig. 4B and data not shown). In contrast, extracts derived from the *sufA* mutant CP1223 showed wild type levels of both subunits (Fig. 4B, D). These data indicate that the reason no hydrogenase 1 or hydrogenase 2 activity could be detected in the *iscA* or *erpA* mutants (see Fig. 2) was due to the absence of the small subunit. Moreover, the data suggest that in the ATC mutants the small subunits of both hydrogenases are more rapidly degraded, possibly because they lack a full complement of [Fe-S] clusters.

### The processed catalytic HybC subunit of hydrogenase 2 is not membrane-associated in *erpA* and *iscA* mutants

**Figure 5:**
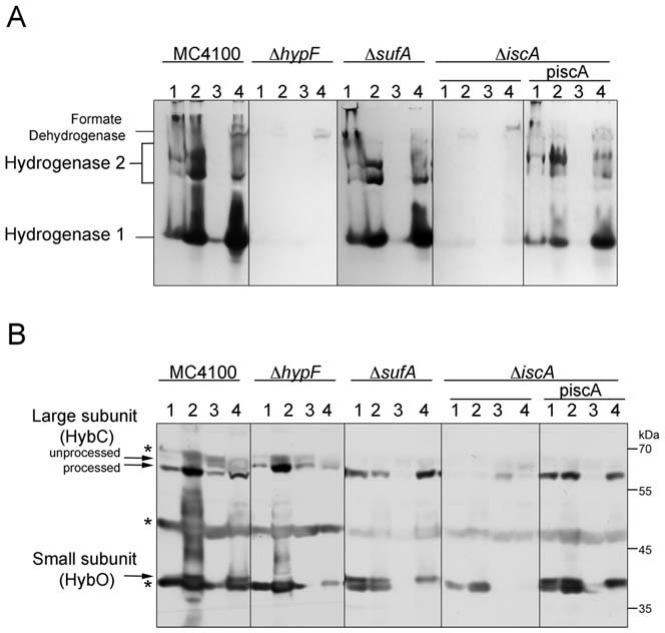
Subcellular localization of hydrogenase 2 in *iscA*, *sufA* and *erpA* mutants. Aliquots (25 µg protein) derived from whole cells (1), crude extracts (2), soluble fractions (3) or membrane fractions (4) from MC4100, DHP-F2 (Δ*hypF*), CP1223 (Δ*sufA*), CP477 (Δ*iscA*) and CP477+piscA were separated either by native-PAGE (A) (7.5% w/v polyacrylamide) and stained for hydrogenase enzyme activity, or by 10% SDS-PAGE (B) and subjected to Western blotting using anti-hydrogenase 2 antiserum. On the left side of panel A, the migration positions of hydrogenase 1, hydrogenase 2, and of the hydrogenase-independent formate dehydrogenase hydrogen: BV oxidoreductase activity are indicated. In panel B the migration positions of the unprocessed and processed forms of the catalytic subunit HybC and the small subunit HybO are shown. The asterisks indicate unspecifically cross-reacting polypeptides of unknown identity, which acted as internal loading controls. The migration positions of the molecular mass standards (in kDa) are indicated on the right of the Figure.

Convincing evidence has been presented to indicate that the hydrogenase small subunit associates with the large subunit only after the [NiFe] cofactor has been inserted [13], [20]. As the small subunits HyaA and HybO of hydrogenases 1 and 2, respectively, bear the signal peptide for recognition and membrane transport to the periplasmic side of the membrane by the Tat translocon, membrane translocation can only take place after complex formation between the mature catalytic subunit and the mature small subunit has occurred. In order to determine how the mutations in the genes encoding the ATC proteins affected the subcellular localization of the catalytic subunits of the hydrogen-oxidizing enzymes and whether they could indeed still be detected, we examined hydrogenase 2 distribution in the soluble and membrane fractions. Crude extracts of the respective mutants were fractionated into soluble and membrane fractions and analyzed by native PAGE, with subsequent activity-staining, and by western blotting with anti-hydrogenase 2 antiserum (Fig. 5). In extracts derived from MC4100, both hydrogenase 1 and hydrogenase 2 activities were associated with the membrane fraction (Fig. 5A), which was expected and served as a positive control [22], [23], [31]. Strain DHP-F2 (*hypF*) lacks hydrogenase activity [26] and acted as a negative control (Fig. 5A). The weak, slowly migrating hydrogen: benzyl viologen oxidoreductase activity observed in the membrane fraction of strain DHP-F2 was due to the side-activity of formate dehydrogenase [25].

Western blot analysis demonstrated that the processed form of the hydrogenase 2 large subunit, HybC was also found associated with the membrane fraction (Fig. 5B). In contrast, after fractionation of extracts from CP477 (Δ*iscA*), the low amount of processed HybC polypeptide still detectable was primarily found in the soluble fraction (Fig. 5B) and no hydrogenase 2 activity was associated with this material (Fig. 5A). Fractionated extracts derived from the *sufA* mutant CP1223 showed a similar distribution of enzyme activity (Fig. 5A) and the catalytic subunit, HybC, (Fig. 5B) to that in extracts of MC4100. While a clear membrane association of the HybO small subunit of hydrogenase 2 was observed in fractionated extracts of MC4100 and the *sufA* mutant (Fig. 5B), no HybO could be detected in any sub-cellular fraction, or in whole cells of the *iscA* mutant CP477 or indeed the *hypF* mutant DHP-F2.

Introduction of the *iscA* gene on plasmid piscA into CP477 restored the activities of both hydrogen-oxidizing enzymes, their association with the membrane fraction (Fig. 5A), as well as the appearance of the membrane-associated, processed HybC large subunit and the small subunit HybO (Fig. 5B).

Similar results to those observed for CP477 (Δ*iscA*) were also observed when the sub-cellular fractions of the *erpA* mutant LL401 were analyzed (data not shown). Taken together, these results demonstrate that in the absence of the ATC proteins IscA or ErpA the small subunit of hydrogenase 2 could not be detected in any sub-cellular fraction. The lack of a small subunit results, presumably, in more rapid degradation of the processed, matured large subunit and the remaining detectable polypeptide was mainly soluble.

### The [Fe-S] cluster-containing maturase HypD is present in *erpA* and *iscA* mutants but not in a strain lacking the scaffold protein IscU

**Figure 6:**
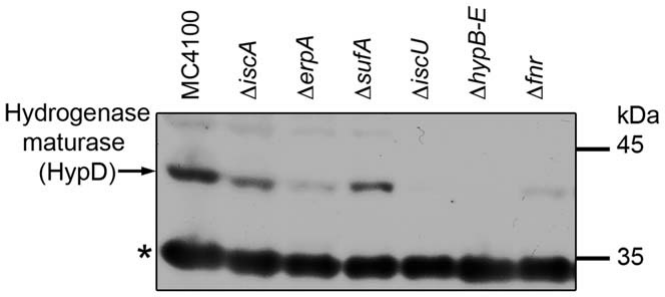
Identification of the HypD maturase in mutants defective in iron-sulphur cluster biogenesis. Aliquots (25 µg protein) derived from MC4100, CP477 (Δ*iscA*), LL402 (Δ*erpA*), CP1223 (Δ*sufA*), JW2513 (Δ*iscU*), BEF314 (Δ*hypB-E*) and PB1000 (Δ*fnr*) were separated by 10% (w/v polyacrylamide) SDS-PAGE and subjected to western blotting using anti-HypD antiserum. The asterisk indicates an unidentified cross-reacting polypeptide that served as an internal loading control. The migration positions of the molecular mass standards (in kDa) are indicated on the right of the figure.

The [4Fe-4S] cluster-containing HypD maturase, together with a number of other maturase enzymes, is required for the biosynthesis of the [NiFe] cofactor in the large subunit of [NiFe]-hydrogenases [32]–[34]. It was reported [13], [33] that amino acid exchanges in a quartet of the C-terminally localized Cys residues in HypD, which coordinate the [4Fe-4S] cluster [34], destabilize the protein. Cell-free, crude extracts derived from MC4100 and the [Fe-S] cluster-trafficking mutants were separated by SDS-PAGE and subjected to western blot analysis with anti-HypD antibodies (Fig. 6). HypD migrated as an approximately 40 kDa polypeptide in extracts from MC4100. An extract derived from the *hyp* operon deletion mutant BEF314 [35] showed no polypeptide that migrated at this position or that reacted with the anti-HypD antibodies (Fig. 6). Expression of the *hyp* operon is regulated by the [Fe-S]-containing regulator Fnr [36] and extracts of the *fnr* mutant PB1000 [27] showed reduced levels of the Fnr HypD protein (Fig. 6). While extracts from CP477 (Δ*iscA*) and CP1223 (Δ*sufA*) had levels of HypD essentially similar to those in MC4100, an extract derived from an *erpA* mutant showed a HypD polypeptide of reduced intensity. In contrast, extracts from the *iscU* mutant JW2513 showed no polypeptide, a result which would be consistent with a lack of the [Fe-S] cluster in HypD causing rapid turnover of the protein (see [13]). This result demonstrates that the Isc machinery is also required for insertion of the [Fe-S] cluster into HypD, but that in contrast to the hydrogenase small subunits, IscA and ErpA show redundancy in cluster delivery to HypD.

## Discussion

### Deficiences in anaerobic modular [Fe-S] cluster enzyme assembly

In this study we have shown that a mutant lacking the A-type carrier proteins ErpA and IscA cannot oxidize molecular hydrogen. A mutant unable to synthesize the [Fe-S] cluster scaffold protein IscU of the Isc machinery [3] was also deficient in [NiFe]-hydrogenases 1 and 2. Both IscA and ErpA are therefore essential for synthesis of functional hydrogen-oxidizing hydrogenases. The severe reduction in overall hydrogenase enzyme activity, measured as hydrogen-dependent BV reduction, and the complete loss of hydrogenase 1 and hydrogenase 2 activities, suggests that IscA and ErpA are also probably important for hydrogen-evolution by *E. coli*, which is catalyzed by hydrogenase 3 [23], [37]. The lack of an effect of a *sufA* mutation on hydrogenases 1 and 2 rules out any direct involvement of the SufA protein in anaerobic hydrogen oxidation and underscores and extends the genetic evidence indicating that IscA and ErpA are solely involved in the biogenesis of [Fe-S] clusters during anaerobic respiratory metabolism [3]. The findings of the current study are also supported by the recent demonstration that the modular anaerobic respiratory enzymes formate dehydrogenase and nitrate reductase are inactive in *erpA* mutants and severely reduced in *iscA* mutants [38]. Taken together these data indicate that the Isc machinery is crucial for the biosynthesis and assembly of functional modular anaerobic oxidoreductases that depend on [Fe-S] clusters for electron transfer processes.

### Absence of the small subunit is the reason for the lack of hydrogenase enzyme activity

The lack of hydrogenase 1 and 2 enzyme activity (measured as benzyl viologen: oxidoreduction) in the individual ATC mutants proved to be due to the absence of the small, electron-transferring subunit, which relays electrons between the catalytic site in the large subunit and the quinone pool [15], [39], [40]. In the respiratory hydrogenase 1 and 2 enzymes each small subunit has three [Fe-S] clusters with the medial one being a [3Fe-4S] cluster flanked by [4Fe-4S] clusters in the case of hydrogenase 2 and by a [4Fe-4S] and a [4Fe-3S] cluster in the case of hydrogenase 1 [40], [41]. We believe it is valid to assume that if the [Fe-S] clusters cannot be inserted into these proteins they are rapidly degraded. This is strongly supported by the apparent instability of the [Fe-S] cluster-containing [NiFe]-hydrogenase maturase HypD observed previously, which was caused by substitution of the Cys residues that coordinate the cluster [33] and the complete absence of HypD in an *iscU* mutant shown in this study (see Fig. 6). Our recent demonstration that in the absence of the small subunit hydrogen-dependent benzyl viologen reduction is abolished [24] explains why enzyme activity could not be detected in the ATC mutants.

Because the HyaA and HybO small subunits of hydrogenases 1 and 2, respectively, carry Tat signal peptides [42], if they are rapidly degraded then they are unavailable to associate with the mature catalytic subunit and thus the HyaA-HyaB and HybO-HybC dimeric complexes cannot be translocated by the Tat translocon. Our ability to detect the mature forms of the HyaB and HybC large subunits, albeit in drastically lower amounts compared with MC4100, supports the evidence [20], [42] that the matured large subunits of hydrogenase 1 and hydrogenase 2 become trapped in the cytoplasmic compartment. Moreover, because the processed form of the HybC large subunit of hydrogenase 2 could be detected in the soluble fraction this indicates that biosynthesis of the [NiFe] cofactor was not compromised in the ATC mutants. This provides futher evidence that the large subunit is matured independently of the small subunit. The findings also indicate that both proteins form a complex only after maturation of each has been completed.

The detection of processed, mature forms of the catalytic subunits of hydrogenases 1 and 2 in the ATC mutants indicates that delivery of the iron for biosynthesis of the [NiFe] cofactor is either independent of the [Fe-S] cluster biogenesis machinery, or that the IscA and ErpA proteins show redundancy in this function. Because the [Fe-S] cluster-containing HypD maturase could be detected in both ATC mutants but not in an *iscU* mutant this indicates that the [Fe-S] cluster in HypD relies upon the Isc machinery and IscA and ErpA exhibit redundancy in this insertion process. Unfortunately, the question of whether the ATC proteins might be involved in Fe delivery for [NiFe] cofactor biosynthesis cannot be resolved by the findings presented in this study because without HypD it is not possible to synthesize and introduce the [NiFe] cofactor into the hydrogenase catalytic subunits. This problem will require the development of an *in vitro* system [43] to address the origin of the iron atom in the [NiFe] cofactor.

### Do ErpA and IscA deliver different [FeS] clusters to apoprotein targets?

The dependence on both IscA and ErpA for [Fe-S] cluster insertion into the small subunits of hydrogenases 1 and 2 in *E. coli* suggests different roles for these proteins in cluster insertion. This conclusion has also been reached for certain [Fe-S] cluster-containing enzymes involved in aerobic growth [3], [8]. Although the approximately 110 amino acid ErpA and IscA proteins are phylogenetically related, they share only 40% amino acid sequence identity (57% similarity), which would be consistent with them having different apoprotein substrates or cluster assembly functions. Py and Barras have proposed that in aerobically growing *E. coli* IscA accepts a pre-formed [Fe-S] cluster from IscU and transfers this cluster via ErpA to the apo-protein substrate [3]. This model would potentially be consistent with the findings observed for hydrogenases 1 and 2; however, [Fe-S] cluster transfer from IscA to ErpA cannot explain the phenotype of the *iscA* and *erpA* mutations with respect to HypD stability, which suggests some redundancy between the proteins. Rather, it is possible that ErpA might preferentially transfer [4Fe-4S] clusters while IscA might transfer either [4Fe-4S], [3Fe-4S] or [4Fe-3S] clusters. The recent exciting discoveries of a new type of [4Fe-3S] cluster in the small subunit of the oxygen-tolerant, membrane-associated respiratory [NiFe]-hydrogenase in *Ralstonia eutropha* [44], *Hydrogenovibrio marinus* [45] and hydrogenase 1 of *E. coli* [41], together with the findings presented here, clearly indicate that either IscA or ErpA performs this function.

### The paradoxical phenotype of a *hypF* mutant and the spatio-temporal assembly of hydrogenases

HypF is central to the maturation of [NiFe]-hydrogenases because it synthesizes the CN^−^ ligands to the Fe atom, which is present in the [NiFe] cofactor of the catalytic subunit of [NiFe]-hydrogenases [13]. The consequence of deleting the *hypF* gene is that the catalytic subunits do not receive the [NiFe] cofactor, consequently remain unprocessed and are enzymatically inactive. The results of this study demonstrate that the small subunits of hydrogenase 1 and hydrogenase 2 cannot be detected in a *hypF* mutant, which is a similar phenotype to that observed in Isc^−^ mutants. While in *iscA* and *erpA* mutants the residual large subunit is processed and mature, in the *hypF* mutant the large subunit remains unprocessed. Surprisingly, if the large subunit cannot be matured the small subunit appears to be rapidly degraded. Equally, if the small subunit does not receive its complement of [Fe-S] clusters then it is rapidly degraded and the processed large subunit is then also degraded, albeit more slowly. The apparent paradox lies in the observation that the unprocessed large subunit is clearly comparatively stable in a *hypF* mutant but the processed species is not stable in an *iscA* or *erpA* mutant (see Fig. 4). This suggests that the unprocessed catalytic subunit is somehow stabilized against degradation. The molecular basis underlying this stability is currently unclear.

Based on the findings presented here and those of previous studies from other groups, the temporal order of events along the maturation pathway of the hydrogen-oxidizing hydrogenases can be summarized as follows. First, IscA or ErpA delivers the [Fe-S] cluster to apo-HypD and together with the other Hyp maturases [13] these proteins synthesize the [NiFe] cofactor and insert it into the precursors of the HybC and HyaB large subunits. Whether the Fe atom in the active site cofactor is derived from the Isc machinery or is delivered by another route remains to be elucidated. Subsequent to processing of the catalytic subunit by removal of the C-terminal peptide [13], maturation of the large subunit is completed. In a presumably independent, but temporally orchestrated, reaction the apo-HybO and apo-HyaA small subunits receive their complement of [Fe-S] clusters from the combined actions of IscA and ErpA. Whether further specific chaperones are involved in this process, as has been suggested from studies on the small subunit of the [NiFe]-hydrogenase in the nitrogen-fixing symbiotic bacterium *Rhizobium leguminosarum* [46], also remains to be established. Once maturation has been completed the holo-form of the small subunit can associate with the mature large subunit to form the holo-dimer that is a proficient substrate for the Tat translocon. If the [Fe-S] clusters cannot be inserted into the small subunit it is directed to the protein degradation machinery.

## Methods

### Strains, plasmids and growth conditions

All bacterial strains and plasmids used in this study are listed in Table 1.

**Table 1:** Strains and plasmids used in this study

Strains/plasmids	Genotype^a^	Reference/Source
MC4100	F^−^ *araD139* Δ(*argF-lac*)*U169 ptsF25 deoC1 relA1 flbB5301 rspL150* ^−^	[53]
BEF314	MC4100 Δ*hypB-hypE2 (hyp:: cat* pACYC184)	[35]
DHP-F2	MC4100 Δ*hypF* 59-629AA; ECK2707	[26]
PB1000	MC4100 Δ*purT* Δ*purU* Δ*insH4-fnr*	[27]
CP795	MC4100 Δ*hyaB hybO hycE*	[24]
CP477	As MC4100 but Δ*iscA*::Kan^R^; ECK2525	[38]
CP1223	As MC4100 but Δ*sufA*::Cm^R^; ECK1680	[38]
LL401	MG1655 conditional *ara_P_*::*erpA*	[8]
LL402	MG1655 Δ*erpA*::Cm^R^	[8]
JW2513	BW25113 Δ*iscU*::Kan^R^; ECK2526	+
Plasmids		
pCP20	*FLP* +, λcI857+, λ*p*_R_ Rep^ts^, Amp^R^, Cm^R^	[49]
perpA	pBluescript SK(+) containing *erpA* in BamHI and EcoRI site; Amp^R^	[38]
piscA	pBluescript SK(+) containing *iscA* in BamHI and EcoRI site; Amp^R^	[38]

+ National BioResources Project (NIG, Japan): E. coli.

a Allele numbers are given for single gene mutants and refer to the K-12 nomenclature.

*E. coli* was grown aerobically in Erlenmeyer flasks filled to maximally 10% of their volume with TGYEP medium [47]. Cultures were incubated on a rotary shaker (250 rpm) and at 37°C. Anaerobic growths were performed at 37°C in sealed bottles filled with anaerobic TGYEP medium under a nitrogen gas atmosphere. When required, the growth medium was solidified with 1.5% (w/v) agar. All growth media were supplemented with 0.1% (v/v) SLA trace element solution, including 7.5 µM iron chloride [48]. The antibiotics chloramphenicol, kanamycin and ampicillin, when required, were added to the medium at the final concentrations of 12.5 µg ml^−1^, 50 µg ml^−1^ and 100 µg ml^−1^, respectively. Where indicated, L-arabinose was added to the growth medium to 0.2% (w/v).

When required, the Kan^R^ cassette of certain mutants was removed by transforming the strain in question with pCP20 encoding a Flp-recombinase [49]. Mutants were subsequently tested for sensitivity to kanamycin.

### Preparation of cell extracts and determination of enzyme activities

Anaerobic cultures were harvested at an OD_600 nm_ of approximately 0.8. Cells from cultures were harvested by centrifugation at 4000× g for 10 min at 4°C, resuspended in 2–3 ml of MOPS buffer pH 7.0 and lysed on ice by sonication (30 W power for 5 minutes with 0.5 sec pulses). Unbroken cells and cell debris were removed by centrifugation for 15 min at 10 000× g at 4°C and the supernatant was used as the crude cell extract. Membrane and soluble fractions were prepared as described [22]. Samples designated as cells in the figures indicates that whole cell samples were collected by centrifugation and the cell pellets were either resuspended directly in SDS sample buffer for western blot analysis or they were treated with 4% (v/v) Triton X-100 for 30 min on ice prior to being loaded directly onto non-denaturing polyacrylamide gels in 100 µl of the respective buffer at an optical density OD_600 nm_ equivalent to 1. Protein concentration of crude extracts was determined [50] with bovine serum albumin as standard. Hydrogenase activity was measured according to [22] except that the buffer used was 50 mM MOPS, pH 7.0. The wavelength used in the hydrogenase enzyme assay was 578 nm and an E_M_ value of 8,600 M^−1^ cm^−1^ was assumed for reduced benzyl viologen. One unit of activity corresponded to the oxidation of 1 µmol of hydrogen per min. Experiments were performed minimally three times and each time enzyme assays were perfomed in triplicate. Data are presented as standard deviation of the mean.

### Polyacrylamide gel electrophoresis and immunoblotting

Aliquots of 25–50 µg of protein from the indicated sub-cellular fractions were separated by SDS-polyacrylamide gel electrophoresis (PAGE) using 10% (w/v) polyacrylamide [51] and transferred to nitrocellulose membranes as described [52]. Antibodies raised against hydrogenase 1 (1∶ 10000; [31]), hydrogenase 2 (1∶20000; a kind gift from F. Sargent) and HypD (1∶3000; a kind gift from A. Böck) were used. Secondary antibody conjugated to horseradish peroxidase was obtained from Bio-Rad. Visualisation was done by the enhanced chemiluminescent reaction (Stratagene).

Non-denaturating PAGE was performed using 5% (w/v) polyacrylamide gels pH 8.5 and included 0.1% (w/v) Triton X-100 in the gels and running buffer [22]. Samples (25 µg of protein) were incubated with 5% (w/v) Triton X-100 prior to application to the gels. Hydrogenase activity-staining was done as described in [22] except that the buffer used was 50 mM MOPS pH 7.0.
